# Effects of litter floor access and inclusion of experienced hens in aviary housing on floor eggs, litter condition, air quality, and hen welfare

**DOI:** 10.3382/ps/pey525

**Published:** 2018-12-11

**Authors:** Jofran L Oliveira, Hongwei Xin, Lilong Chai, Suzanne T Millman

**Affiliations:** 1 Department of Agricultural and Biosystems Engineering, Iowa State University, Ames 50011-3270, IA, USA; 2 Egg Industry Center, Ames 50011, IA, USA; 3 Department of Poultry Science, University of Georgia, Athens 30602, GA, USA; 4 Department of Veterinary Diagnostic & Production Animal Medicine, Iowa State University, Ames 5011-1134, IA, USA

**Keywords:** food safety, poultry management, animal welfare, air quality

## Abstract

With different cage-free (CF) housing styles and management schemes, retailers have developed their own CF criteria. One highly debated aspect is if hens may be kept inside the system for part of the day—during the first few hours after lights-on. Research is lacking regarding the impacts of such a practice on hen welfare, incidence of eggs laid on the litter floor, litter condition, and air quality. This 14-mo field study was conducted to help assess such impacts. Hens (Dekalb White) in an aviary house (50,000-hen nominal capacity) were allowed to have full litter access (FLA) vs. part-time litter access (PLA) from 10:50 am to 9:00 pm, coupled with the absence or presence of experienced hens (1.5% of the population), hence a 2 × 2 factorial arrangement. The measured variables included a) incidence of floor eggs, b) percentage of birds remaining on litter floor at night, c) mortality, d) body weight (BW) and BW uniformity, e) litter condition (depth, moisture content, texture, amount removed, and bacteria concentration), f) environmental conditions, and g) welfare conditions (10 variables). Compared to FLA, PLA had a significantly lower incidence of floor eggs (1.4 ± 0.1 vs. 12.6 ± 1.1 eggs per hen housed as of 76 weeks of age (WOA), i.e., approximately 89% reduction), less manure deposition on the floor (0.53 ± 0.02 vs. 1.05 ± 0.04 kg/100 hens/d, dry basis, i.e., approximately 50% reduction), and lower ammonia concentrations due to drier litter (averaging 22% lower). Inclusion of 1.5% experienced hens in the young flock did not show benefit of reducing the incidence of floor eggs (*P* = 0.48). The percentage of hens remaining on the floor at night was low (< 0.01%) in all cases from 24 WOA onward. No differences were detected between FLA and PLA in hen welfare conditions, mortality, BW, BW uniformity, bacteria concentration in the litter, air temperature, or relative humidity.

## INTRODUCTION

Cage-free (**CF**) egg production has been a topic of increasing importance in the United States due to pledges made by food retailers and restaurants to source only CF eggs by certain year (e.g., 2025) (Chai et al., 2018a). However, issues or challenges associated with CF production remain to be addressed with regard to food quality and safety (Hannah et al., 2011; Holt et al., 2011; Jones et al., 2015), litter usage and dust bathing motivation (Colson et al., 2007; Ali et al., 2016; Campbell et al., 2016a), indoor air quality and emissions (Zhao et al., 2013, 2015, 2016), and welfare (Blatchford et al., 2016; Widowski et al., 2016; Louton et al., 2017). With different CF styles and management schemes, retailers have developed their own CF criteria (Mench et al., 2011; Scott et al., 2017). One of the debated criteria is concerning litter access, namely whether the laying hens should be provided full litter access throughout the day to be qualified as CF egg production, as compared to temporarily confining the hens in the aviary system (one type of CF system) during oviposition period in early morning. Research is lacking regarding the impact of such practice on hens’ welfare, floor eggs, litter, and environmental conditions.

One of the main improvements of CF systems is the inclusion of litter floor area, allowing the hens to express their natural behavior of dust bathing (Colson et al., 2007). In previous studies, laying hens performed dust bathing throughout the day, but the activity peaked in late morning and afternoon (Vestergaard, 1982; Hansen, 1994; Campbell et al., 2016a). In the morning, the hens’ priority is the pre-laying and laying motivation (Hunniford et al., 2014, 2017); depending on where the eggs are nested the hens can be classified as nest layers or floor layers (Sherwin and Nicol, 1993; Cooper and Appleby, 1996; Kruschwitz et al., 2008; Zupan et al., 2008). Oviposition place is one of the biggest concerns in CF systems because floor eggs are directly linked to food safety and economic issues (De Reu et al., 2008; Jones et al., 2015). Therefore, it is essential to develop and test strategies to reduce the incidence of floor eggs in commercial CF systems.

Previous studies have demonstrated that animals exposed to the behavior of a trained demonstrator subsequently acquire the relevant novel response more rapidly; however, social factors have an important influence in determining whether social learning will occur (Nicol and Pope, 1992, 1994). From this perspective, it may be worthy of investigating if hens experienced in laying eggs in the colony nest in aviary systems can help training or motivating the novice young hens to use the colony nests.

The inherent feature of litter area access in CF production systems is that part of the hen manure is deposited on the floor and remain on it for an extended period, as compared to 100% of the manure deposited onto the manure belt underneath each cage tier and frequently removed from houses. The end result is a much less desirable indoor air quality for both the animals and the caretakers and elevated air emissions (Jones et al., 2015; Zhao et al., 2015; Chai et al., 2018a). Then it is reasonable to expect that managing litter access by the hens or the amount of manure deposition on the litter floor can be conducive to improving the litter conditions and indoor air quality. On the other hand, there is a general concern that limiting litter access of the hens would affect expression of animal's natural behaviors, which may lead to compromised welfare (Alm et al., 2015). There has also been anecdotal claim that confining hens inside the systems negatively affect flock uniformity. However, data are lacking to substantiate the concerns or claims.

Therefore, the objective of this study was to evaluate the effects of full litter access (**FLA**) vs. part-time litter access (**PLA**) and inclusion of experienced hens or not on occurrence of floor eggs, litter condition, indoor air quality, and hen welfare through a long-term field study involving a commercial aviary hen housing system. The hypotheses were that a) PLA would be beneficial in reducing floor eggs, improving litter condition on the floor and thus ammonia generation while not adversely affecting mortality, body weight (BW) and BW uniformity, or welfare of the hens; and b) inclusion of 1.5% experienced birds would be conducive to training a young flock of hens in their nesting behavior, hence reducing incidence of floor eggs.

## MATERIALS AND METHODS

### Animals and Housing

An aviary CF henhouse (153 m L × 21 m W × 3 m H) with concrete floor and containing Big Dutchman Natura 60™ aviary system was used in this field study, initially housing 51,405 Dekalb White pullets at 17 weeks of age (**WOA**). The pullets had been beak trimmed at hatchery and reared in an aviary pullet house.

The aviary house featured system doors that could be controlled to stay open or closed. The henhouse was comprised of 40 sections, of which 32 were enrolled in the 4 experimental treatments (8 sections per treatment). A total of 16 outer sections (15 m L × 3 m W × 3 m H) were located adjacent to the sidewalls of the house, each containing 857 birds. A total of 16 inner sections (15 m L × 6 m W × 3 m H) were located in the interior of the house, each containing 1,714 birds. Hence, approximately 10,280 pullets were allocated to each treatment balanced over 4 inner sections and 4 outer sections. The stocking density was 525 cm^2^ hen^−1^ on the litter floor and 620 cm^2^ hen^−1^ in the aviary system.

The 4 experimental treatments were as follows: 1) FLA with pullets only (i.e., absence of experienced hens) (**FLA_P_**), 2) FLA with pullets plus 1.5% experienced hens (**FLA_E_**), 3) PLA (10:50 am to 9:00 pm per day) with pullets only (**PLA_P_**), and 4) PLA with pullets plus 1.5% experienced hens (**PLA_E_**). The lighting program ranged from 12 to 16 h depending on the hens’ age. After 24 WOA, the light came on at 5:00 am and started going off at 9:00 pm, with a 45-min dimming period. In this paper, the word “regimen(s)” is used when comparing the effect of litter access (PLA vs. FLA), whereas the word “treatment(s)” is used when evaluating the effect of litter access nested with the inclusion of experienced hens or not (FLA_P_, FLA_E_, PLA_P_, and PLA_E_).

Upon transfer to the laying house, all pullets were kept inside the system for 10 d to ensure familiarity with the system (e.g., location of feed, water, and the colony nest) before starting the respective treatments. The entire floor area of the house was initially covered with approximately 340 kg of wood shavings, uniformly distributed with 7 kg in each of the outer sections and 14 kg in each of the inner sections, before the litter access was provided. After day 10, the system doors in the FLA regimen were opened and remained open, and experienced hens (1.5% population) were introduced to the FLA_E_ treatment. Hens in the PLA regimen followed the management practice of being kept inside the system for a total of 4 wk to ensure that they would start laying eggs in the colony nest before having access to the litter floor area. At 22 WOA, the PLA birds were allowed daily access to the litter floor from 10:50 am (after the general oviposition time) until lights-off (9:00 pm), and experienced hens (1.5% population) were introduced to the PLA_E_ treatment. For convenience and biosecurity, experienced hens were obtained from another aviary house on the same farm; experienced hens were Bovan Whites at 49 and 53 WOA when enrolled in the FLA_E_ and PLA_E_ treatments, respectively. Feed was provided to the hens, via feed conveyor chains, 4 times a day at 05:30 am, 09:30 am, 2:30 pm, and 4:30 pm.

### Measurements and Data Collection

#### Floor Eggs

The number of floor eggs was counted manually, once a day, and recorded in the checklist provided in each of the 32 sections.

#### Birds on the Floor at Night

The birds remaining on the litter floor after the lights off at night were counted manually, early in the morning (before lights came on), and recorded in the checklist in each section. The measurement was taken daily during the first 3 mo, and weekly afterwards.

#### Mortality

The number of dead birds in each section was counted manually and recorded on the checklist once a day.

#### BW and BW Uniformity

A total of 50 birds per treatment were randomly selected and weighed weekly. Average and standard error were calculated for each of the 4 treatments.

#### Litter Conditions

Litter samples were collected from the litter floor once a month, in 3 different locations (under the system, under the outside perch at the litter area, and in the open litter area) of 16 sections in the henhouse (4 sections per treatment). Litter texture was visually observed once a month in all 32 sections. Litter depth was measured at 3 locations in each of the 32 sections (under the system, under the outside perch at the litter area, and in the open litter area) using a wooden stick and a metal ruler. The wooden stick was inserted into the litter until reaching the concrete floor. A line was drawn on the stick at the litter surface level and the depth measured with the metal ruler. Litter moisture content was determined by oven drying of 10 g litter sample at 105°C for 24 h. The texture of litter area in each section was classified as “loose,” “partially caked” (presence of caking in < 50% of the litter area), and “caked” (presence of caking in > 50% of the litter area). Litter on the floor in both regimens was removed from the aviary house during weeks 37/38, 54/55, and 77/78 due to excessive accumulation in the FLA regimen, and the amount of litter removed (volume and weight) was determined and recorded. Litter samples were tested for bacteria concentration at the end of the experiment. A composite litter sample (approximately 50 g) representing 3 locations of each section (under the system, under the outside perch at the litter area, and in the open litter area) was collected by scooping the litter, and this was done for 16 sections (i.e., 4 sections per treatment). The 16 collected samples were transported in an ice-chest cooler to the analytical laboratory at Iowa State University, where 1 g of litter (as is) from each sample was transferred into 9 mL physiological saline solution, and homogenized by vortexing for 30 s and serially diluted (1:8). Viable counts of total bacteria were determined by plating 0.1 mL portions onto plates of trypticase soy agar (Catalog No. R455002, Fisher Scientific, Hanover Park, IL). The plates were aerobically incubated at 37°C for 24 h. The colonies formed on plates (30 to 300 colonies) were counted and used for calculating bacteria concentration by the following equation (Eq. 1) (Zhao et al., 2016; Chai et al., 2018b):

(1) \begin{equation*} {\rm{BC\ }} = lo{g_{10}}\left( {{\rm{\ }}\frac{{n{\rm{\ }} \times {\rm{\ }}{{10}^d}}}{{{V_{\rm{p}}}}}{\rm{\ }} \times {\rm{\ }}{V_{\rm{s}}}} \right) \end{equation*}

where BC is the bacteria concentration (logCFU/g), CFU is colony-forming unit; *n* is the number of colonies found in the plate (30 to 300 colonies), *V*_p_ is the volume of portion plated (mL), *d* is the serial dilution factor (0 for undiluted sample and 1 for 10-fold diluted sample, etc.), and *V*_s_ is the total volume of original liquid sample (mL).

#### Environmental Conditions

Ammonia (NH_3_) concentration (ppm) was measured at the litter perch level by using 3 different instruments: 1) detection tubes used with a hand pump (RAE Systems^®^, Sunnyvale, CA), 2) electrochemical NH_3_ detector (Honeywell^®^, Sunnyvale, CA), and 3) electrochemical NH_3_ detector (MSA Altair^®^, Mine, WV). The detection tubes were used in 8 sections (2 per treatment), whereas the electrochemical detectors were used in all 32 sections. Air temperature, relative humidity (**RH**), and carbon dioxide (CO_2_) concentrations were continuously monitored and recorded at 10-min intervals with 11 portable T/RH data loggers (Hobo^®^ MX2301, Onset, Bourne, MA) and 2 T/RH/*CO_2_* loggers (Hobo^®^ MX1102, Onset, Bourne, MA). The loggers were placed inside the system (6), in the litter area (6), and outside the aviary house (1).

#### Welfare Status

Welfare assessment of the hens was performed at 72 WOA, with 200 hens randomly selected from 20 sections (5 sections per treatment, 10 hens per section). Methodology for the welfare assessment was adapted from the procedures of Welfare Quality ^®^ Assessment Protocol for Poultry (Welfare Quality^®^, 2009). The assessment method was applied to individual hens rather than at the flock level. Two professionals who were versed in poultry welfare assessment and blind to treatment allocations simultaneously evaluated 5 hens in each of the 20 sections of the house (5 hens × 20 sections = 100 hens/assessor × 2 assessors = 200 hens). Within each section, research personnel randomly selected 5 hens from the litter area and 5 hens from inside the aviary system to present to the assessors for evaluation; number of hens that each assessor evaluated from litter and aviary locations was balanced over the 20 sections.

Each hen was evaluated according to the following welfare characteristics. *Plumage damage* was scored at a maximum of 14 points (sum of the scores from the plumage of head, neck, back, rump, crop, keel, and belly). Each area was scored as 0 (no or slight wear), 1 (moderate wear), or 2 (at least one featherless area ≥ 5 cm in diameter). *Cleanliness* was scored at a maximum of 3 points according to the perceived area of manure soiling on breast, back, rump, belly, and wings, namely 0 (no manure soiling), 1 (slight manure soiling), 2 (moderate manure soiling), or 3 (mostly dirty). *Keel bone deformation* was evaluated via palpation to detect abnormal curvature and scored as 0 if no deformation or 2 otherwise. *Comb pecking wounds* was scored at a maximum of 3 points, i.e., 0 (no evidence of pecking wounds), 1 (1 or 2 pecking wounds), or 2 (3 or more pecking wounds). Comb was also evaluated to investigate signs of *comb abnormality* (blue or black areas, very pale combs, or dried areas). The hens were scored as 0 if no evidence of comb abnormality or 1 otherwise. Foot health was assessed to evaluate *foot pad dermatitis*. The hens were scored at a maximum of 2 points, i.e., 0 (intact feet), 1 (minimal lesion on foot pad), or 2 (visible inflammation, swollen foot). *Toe damage* was also evaluated to investigate signs of wounds or missing parts, i.e., 0 (no toe damage) or 1 otherwise. *Claw length* was scored as 0 if short (< 1 cm) or 1 long (≥ 1 cm). *Lesions in the skin* (wounds not healed) was evaluated and scored as 0 (no lesion), 1 (lesion < 2 cm in diameter), or 2 (lesion ≥ 2 cm in diameter). All hens in this study were beak trimmed; therefore, the condition of *beak trimming* was evaluated and scored as 1 (light to moderate trimming with moderate to no abnormalities) or 2 (severe trimming and clear abnormalities.

The protocols were approved by the Iowa State University Institutional Animal Care and Use Committee (IACUC).

### Statistical Analyses

The sections were considered as the experimental units. Data were tested for homoscedasticity and normality, and transformed when necessary. Mixed model analysis of variance and logistic regression were used to evaluate the effects of litter access management and inclusion of 1.5% experienced hens on the amount of floor eggs, hens on the litter floor at night, mortality, BW, BW uniformity, and welfare status.

The effect of litter access management was further investigated on litter depth, amount of litter on the floor (removed), and litter moisture content and litter bacteria concentration (all using the mixed model), as well as litter texture (using logistic regression model). Litter conditions could in turn affect ammonia generation and thus concentration.

Air temperature, RH, and CO_2_ concentration were evaluated to investigate changes in the microenvironment (inside the system vs. litter area) and in the period of the day (light vs. dark) to observe the homogeneity or heterogeneity inside the aviary housing. The effects of litter access management on temperature and RH were also investigated.

Statistical analysis was performed using JMP 13.2.1 (SAS Campus Drive, Cary, NC). When appropriate, repeated measures analysis was incorporated into the mixed model, and regression analysis was performed. A *P*-value of 0.05 or less indicates a significant difference among the treatments. Unless otherwise specified, data are presented as least squares means along with the standard error of the mean.

## RESULTS

### Floor Eggs

**Figure 1.**
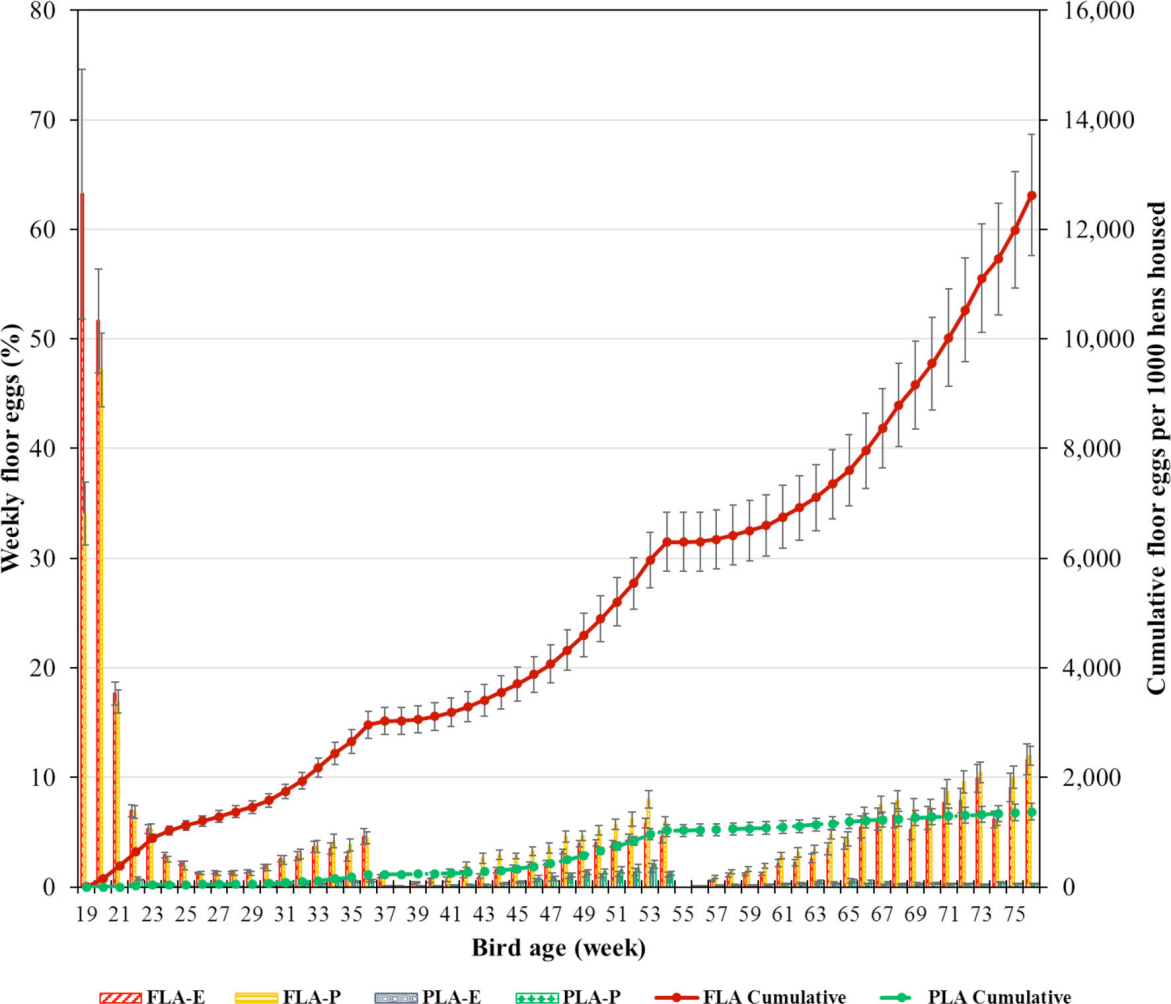
Weekly percentage of floor eggs (bars, %, mean ± SE) and cumulative floor eggs per 1,000 hens housed (lines, %, mean ± SE) of full litter access (FLA) or part-time litter access (PLA) vs. hen age. Hens were kept inside the system during litter removal at 37/38, 54/55, and 77/78 WOA, respectively.

Weekly percentage of floor eggs was not affected by inclusion of 1.5% experienced hens in the young flock (*P* = 0.48), but it was affected by the litter access management (*P* < 0.01). No effect of interaction between inclusion of 1.5% experienced hens and litter access management was found (*P* = 0.54). Overall mean weekly percentage of floor eggs was 4.15 ± 1.53% in FLA and 0.29 ± 0.11% in PLA; 1.05 ± 0.39% with inclusion of 1.5% experienced hens and 1.12 ± 0.42% without. The cumulative floor eggs per 1,000 hens housed as of 76 WOA were 12,625 ± 1,111 and 1,374 ± 148 (i.e., 12.6 ± 1.1 and 1.4 ± 0.1 eggs per hen housed) for the FLA and PLA regimen, respectively (*P* < 0.001). The percentages of weekly and cumulative floor eggs per treatment (mean and SE) are presented in Figure 1. The percentage of floor eggs was higher in the first 2 wk in FLA (> 40%), and reduced gradually until week 25. After the PLA birds had daily litter access (22 WOA), the difference in percentage of floor eggs became evident between the FLA and PLA regimens.

The litter floor cleaning was performed 3 times when hens were 37/38, 54/55, and 77/78 WOA, during which the system was kept closed for all treatments. The abrupt reduction in percentage of floor eggs following the system closure continued after the doors were reopened, with a trend of increase in the subsequent week. Although there was no statistical effect of including experienced hens, FLA_P_ showed a numerically higher percentage of floor eggs than FLA_E_ after the first cleaning period (39 WOA). The overall percentage of floor eggs was 3.33 ± 0.48% in FLA_P_ and 2.74 ± 0.40% in FLA_E_ (*P* = 0.77). The non-significant effect of including the experienced hens was also shown in the PLA regimen, with overall percentage of floor eggs being 0.26 ± 0.04% in PLA_P_ and 0.26 ± 0.04% in PLA_E_ (*P* = 0.99).

### Hens Remaining on Litter Floor at Night

The proportion of birds that stayed outside the system at night was lower than 0.1% and similar for all treatments from 25 WOA onward. Using experienced hens did not affect percentage of hens remaining outside the system at night (*P* = 0.71). However, the proportion of hens outside the system at night was statistically different between the FLA (0.040 ± 0.002%) and PLA (0.010 ± 0.001%) regimens (*P* < 0.001). No significant effect of interaction between inclusion of 1.5% experienced hens and litter access management was found (*P* = 0.85).

### Mortality Rate

**Figure 2.**
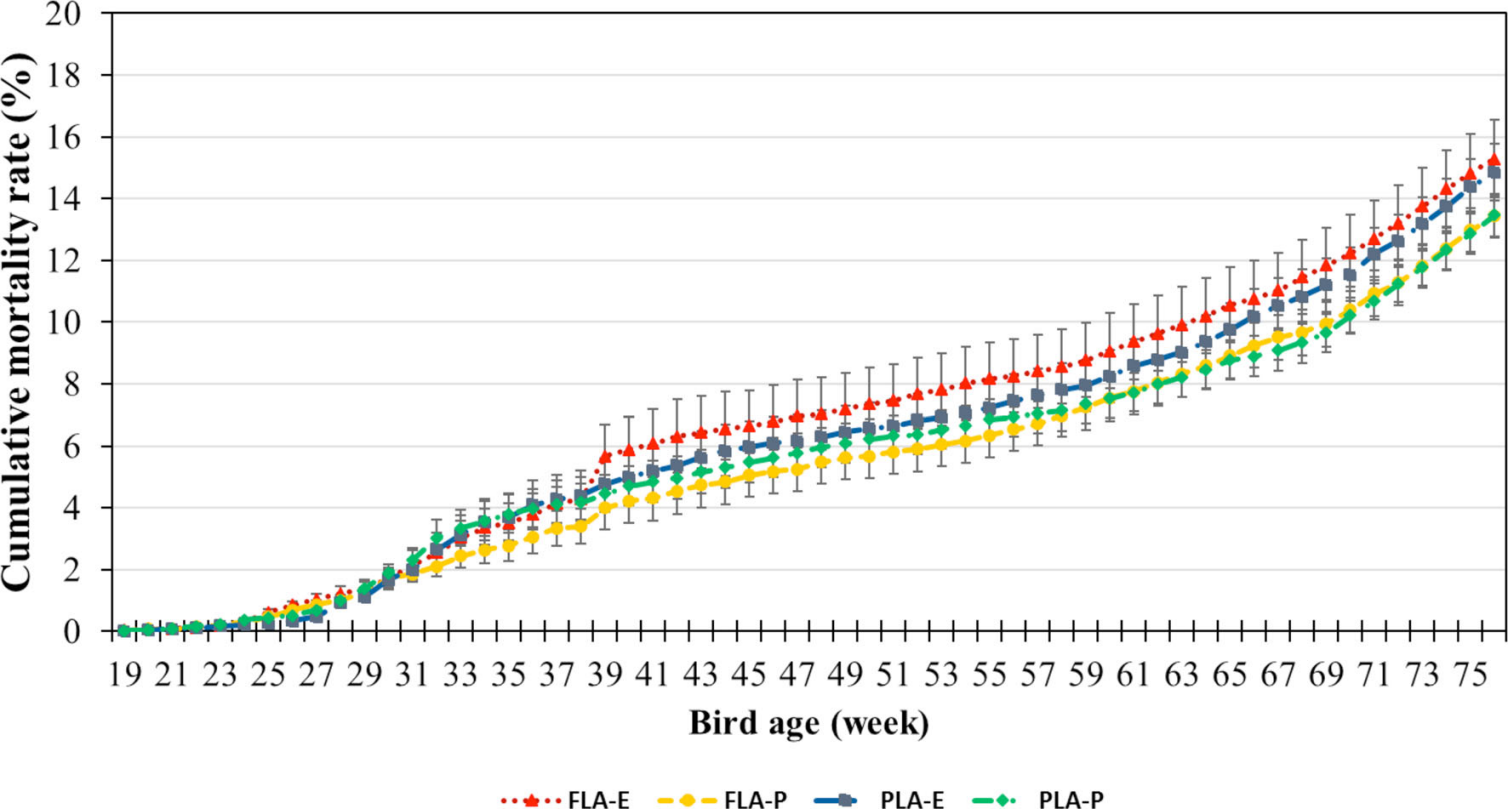
Cumulative mortality (%, mean ± SE) vs. hen age in the 4 different treatments: 1) part-time litter access plus 1.5% experienced hens (PLA_E_), 2) part-time litter access without experienced hens (PLA_P_), 3) full litter access plus 1.5% experienced hens (FLA_E_), and 4) full litter access without experienced hens (FLA_P_).

The weekly and cumulative mortality rates for each treatment are presented in Figure 2. No effect of litter access or experienced hens on mortality rate was observed. Overall weekly mortality rate was 0.22 ± 0.03% in FLA and 0.21 ± 0.03% in PLA (*P* = 0.76), and 0.23 ± 0.03% when including 1.5% experienced hens and 0.21 ± 0.03% when not (*P* = 0.29). No significant effect of interaction between inclusion of 1.5% experienced hens and litter access management was found (*P* = 0.92).

The first litter floor cleaning was performed when hens were 37 to 38 WOA, when the system was kept closed for all regimens. An abrupt increase in mortality rate was observed after the doors were reopened, especially in the FLA_E_ treatment. Due to the difficulty of locating dead birds during the period of cleaning (between 37 and 38 WOA) and a concurrent shortage of caretakers in the same period, it was possible that some of the mortalities incurred during this period were accounted for in the immediate subsequent week record (39 WOA).

### BW and BW Uniformity

BW was not affected by litter access management (1.53 ± 0.01 kg in FLA and 1.51 ± 0.01 kg in PLA, *P* = 0.30) or inclusion of experienced hens (1.52 ± 0.01 kg with experienced hens and 1.53 ± 0.01 kg without, *P* = 0.87). Similarly, BW uniformity was not affected by the litter access management (81.5 ± 0.83% in FLA and 82.9 ± 0.83% in PLA, *P* = 0.17) or inclusion of experienced hens (82.4 ± 0.83% with experienced hens and 82.0 ± 0.83% without, *P* = 0.81). No significant effect of interaction between inclusion of 1.5% experienced hens and litter access management was found for BW (*P* = 0.61) or BW uniformity (*P* = 0.23).

### Litter Conditions

**Figure 3.**
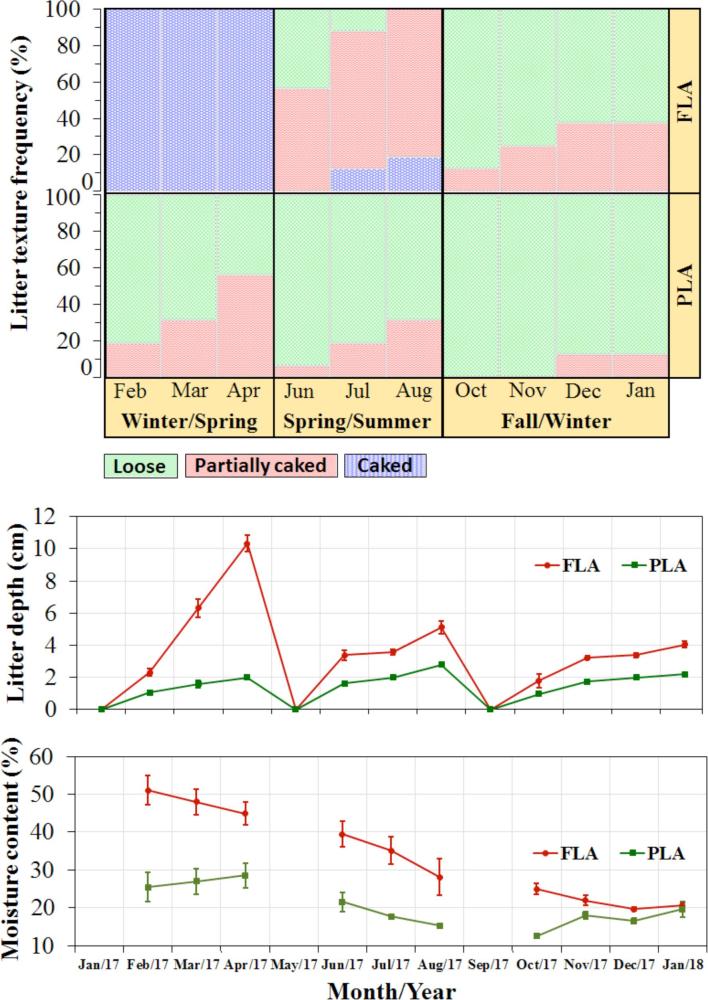
Litter texture (loose, partially caked, or caked), depth (cm, mean ± SE), and moisture content (%, mean ± SE) for the full litter access (FLA) and part-time litter access (PLA) regimens during the experimental period (litter floor was cleaned 3 times: May/17, September/17, and February/18).

Moisture content of the litter was affected by litter access management (31.3 ± 1.6% in FLA and 20.3 ± 1.1% in PLA, *P* < 0.001). Similarly, litter depth was influenced by access to litter area (3.77 ± 0.09 cm in FLA and 1.64 ± 0.04 cm in PLA, *P* < 0.001). The parameters of the litter conditions are summarized in Figure 3.

Litter access management affected the amount of floor litter removed, namely 1.56 ± 0.06 kg/100 hens/d in FLA and 0.67 ± 0.03 kg/100 hens/d in PLA, as-is basis; or 1.05 ± 0.04 kg/100 hens/d in FLA and 0.53 ± 0.02 kg/100 hens/d in PLA, dry basis (*P* < 0.001). However, no effect of the litter access management was observed in the litter bacteria level, 8.98 ± 0.06 logCFU/g in FLA and 8.92 ± 0.06 logCFU/g in PLA (*P* = 0.51).

Litter texture was significantly different between the FLA and PLA regimens (*P* < 0.001). Overall, FLA litter had 33.1% of area in “caked,” 32.5% in “partially caked,, and 34.4% in “loose” category. In comparison, the respective proportions of the PLA litter were 0% “caked,” 18.8% “partially caked,” and 81.3% “loose.”

### Environmental Conditions

Management of litter access had no effect on indoor air temperature (21.7 ± 0.2°C in FLA and 21.7 ± 0.2°C in PLA, *P* = 0.91) or RH (65 ± 1% in FLA and 67 ± 1% in PLA, *P* = 0.34). The location of the hens (system or litter area) did not affect air temperature (21.4 ± 0.2°C in litter and 21.7 ± 0.2°C in system, *P* = 0.20) or RH (66 ± 1% in litter area and 66 ±1% inside the system, *P* = 0.53), but the period of day (light or dark) did affect both temperature (22.6 ± 0.2°C during light period and 20.5 ± 0.2°C during dark period, *P* < 0.01) and RH (64 ± 1% during light period and 67 ± 1% during dark period, *P* < 0.001). There was no statistical evidence of interaction between location (system or litter area) and period of day (light or dark) for temperature (*P* = 0.09) or RH (*P* = 0.15).

**Figure 4.**
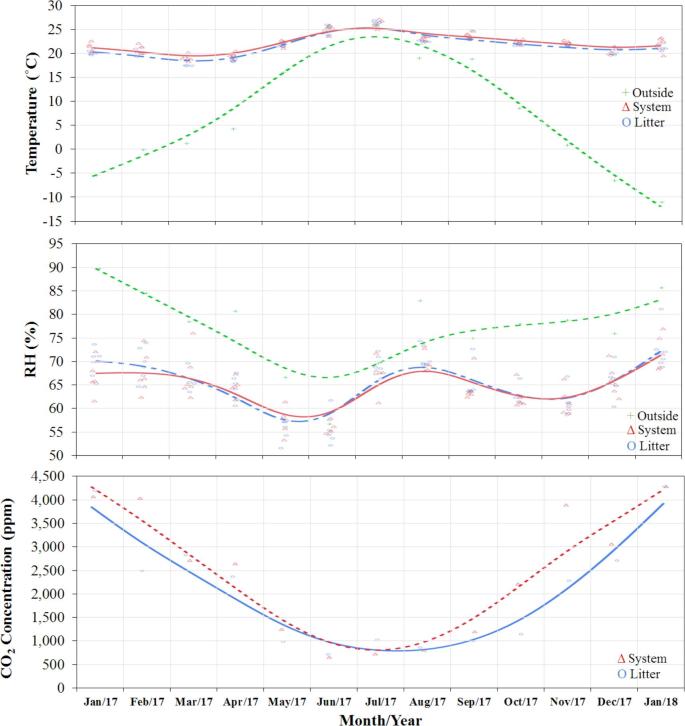
Profiles of air temperature, relative humidity (RH), and CO_2_ concentration of the litter area, inside the system, and outside ambient over the experiment period.

The CO_2_ concentration was 2,372 ± 345 ppm in litter area and 2,034 ± 345 ppm inside the system (*P* = 0.37), and no significant differences were found between light and dark period (*P* = 0.89) or in the interaction between location and period of day (*P* = 0.94). Air temperature, RH, and CO_2_ profiles are shown in Figure 4.

The relationship between indoor CO_2_ concentration (ppm) and ambient temperature (°C), valid for the temperature range –10°C < t < 26°C, can be described by the following empirical model (Eq. 2):

(2) \begin{equation*} {\rm{C}}{{\rm{O}}_2} = 2.504{\rm{\ }}{t^2} - 131.8{\rm{\ }}t + 2577{\rm{\ }}\left( {{\rm{\ }}{r^2} = {\rm{\ }}0.89} \right) \end{equation*}

**Figure 5.**
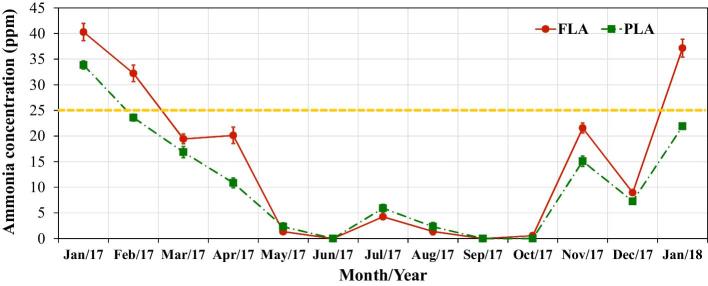
Ammonia concentration (mean ± SE) over the experiment period for the full litter access (FLA) and part-time litter access (PLA) regimens. The horizontal dashed line represents the 8-h exposure threshold for workers recommended by the National Institute for Occupational Safety and Health (NIOSH).

Ammonia concentration was affected by litter access management (17.2 ± 0.8 ppm in FLA and 13.5 ± 0.6 ppm in PLA, *P* < 0.001). Figure 5 shows the seasonal profiles of ammonia concentrations in both regimens.

Ammonia concentration decreased almost linearly from January 2017 (cold weather) to May 2017 (mild weather). The highest ammonia levels mostly occurred in the FLA sections, presumably arising from the more manure accumulation and higher moisture content of the litter. After March 2017, when ventilation rate increased in response to the warmer weather, ammonia concentrations fell below 25 ppm, the 8-h exposure threshold for workers recommended by the National Institute for Occupational Safety and Health (NIOSH) as well as the recommended threshold for poultry housing (NIOSH, 2007).

### Welfare Conditions

During the welfare assessment, panting, piling, enlarged crop, eye pathology, nares discharge or inflammation, enteritis or external parasites were not observed. Data for plumage condition, cleanliness, keel bone deformation, comb pecking, comb abnormality, foot pad dermatitis, claw length, skin lesions, beak trimming, and toe damage are presented in Table 1.

**Table 1.** Mean scores and standard error for the welfare status under the different litter access management and inclusion of experienced hens or not: plumage condition, cleanliness, keel bone deformation, comb peck wounds, comb abnormality, foot pad dermatitis, claw length, skin lesion, beak trimming, and toe damage.

	Litter access (LA)			Experienced hens (EH)			*P-*Value		
Welfare variable	Full	Partial	SE	Yes	No	SE	LA	EH	LA x EH
Plumage condition^1^	4.71	4.97	0.24	4.78	4.90	0.24	0.51	0.62	0.31
Cleanliness^2^	0.43	0.34	0.05	0.35	0.42	0.05	0.33	0.38	0.14
Keel deformation^3^	1.26	1.04	0.10	1.08	1.22	0.10	0.11	0.31	0.64
Comb peck wounds^4^	0.09	0.05	0.03	0.09	0.05	0.03	0.28	0.28	0.54
Comb abnormality^5^	0.01	0.01	0.01	0.01	0.01	0.01	1.00	1.00	0.09
Foot pad dermatitis^6^	0.29	0.38	0.05	0.29	0.38	0.05	0.20	0.20	0.33
Claw length^7^	0.82	0.83	0.04	0.89	0.76	0.04	0.98	**0.01**	0.59
Skin lesions^8^	0.04	0.07	0.02	0.05	0.06	0.02	0.28	0.55	0.28
Beak trimming^9^	1.19	1.18	0.04	1.18	1.19	0.04	0.88	0.88	0.10
Toe damage^10^	0.01	0.02	0.01	0.01	0.01	0.01	0.09	1.00	0.09

^1^Sum of scores from the plumage conditions of head, neck, back, rump, crop, keel, and belly. Each area has a score of 0, 1, or 2 (no wear to moderate and featherless) with a maximum overall score of 14.

^2^Score is 0, 1, 2, or 3 as dirtiness increases.

^3^Score is 0 or 2 for intact or deformed keel bone.

^4^Score is 0, 1, 2, or 3 with increasing evidence of pecking wounds.

^5^Score is 0 or 1 for presence or absence of abnormality.

^6^Score is 0, 1, or 2 with increasing evidence of foot pad dermatitis.

^7^Score is 0 or 1 for short or long claws.

^8^Score is 0, 1, or 2 with increasing evidence of lesions in the skin.

^9^Score is 1 or 2 for moderate or severe trimming.

^10^Score is 0 or 1 for presence of absence of toe damage.

The welfare status was not affected by the litter access management or inclusion of 1.5% experienced hens, with the exception of the claw length condition. Hens with long claws were observed more often in the sections without the experienced hens (*P* = 0.01).

**Figure 6.**
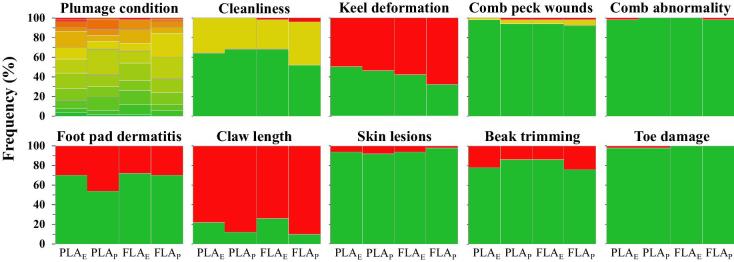
Welfare status of the laying hens in the 4 different treatments: 1) part-time litter access plus 1.5% experienced hens (PLA_E_), 2) part-time litter access without experienced hens (PLA_P_), 3) full litter access plus 1.5% experienced hens (FLA_E_), and 4) full litter access without experienced hens (FLA_P_). The graphs represent the distribution of each welfare aspect, with the color varying from green to red corresponding to best to worst condition, respectively.

Claw length, keel bone deformity, and foot pad dermatitis were the welfare variables with the highest occurrences of the worst conditions. Figure 6 shows the frequency of occurrence of each welfare score per treatment. The green color represents the best welfare status, whereas the red color represents the worst.

## DISCUSSION

In the present study, we performed a comprehensive and novel evaluation of the effect of managing litter floor access and including 1.5% of experienced hens on several production traits, welfare status, litter conditions, air quality, and thermal conditions. The uniqueness of the study lies in its comprehensive and longer-term (entire flock production cycle) nature of the comparative monitoring under field production conditions.

### Floor Eggs

The amount of floor eggs decreased with time in the first 8 wk for all treatments. This trend could be a result of transitioning to stabilization as the birds became more accustomed to using the system structure. Similar result was observed by Cooper and Appleby (1996), who evaluated the incidence of floor eggs from individual laying hens from 22 to 28 WOA and found a reduction from 25 to 5% of eggs laid on the floor, respectively. However, the short period of the Cooper and Appleby study did not allow evaluation of stability of this behavior over the production cycle.

After 28 WOA, we observed a consistent increase in the number of eggs laid on the litter floor for all treatments until the time of floor cleaning when the system was closed for a period of 10 d (Figure 1). During this period, the hens were re-trained to use the system (colony nest); upon allowing for litter area access the percentage of floor eggs was < 1%. In an extensive review on cognition, emotion, and behavior of domestic chickens, Marino (2017) reinforced that learning, particularly in a social context, is an important driver of chicken cognition. But information is scarce about how cognitive abilities play out developmentally into maturity in chickens.

The percentage of eggs laid on the floor increased linearly in the subsequent weeks after the access to litter floor area was re-established. It indicates that the hens’ preference of laying eggs on the floor over the colony nest was not eliminated by the time that they were locked into the system during the cleaning period. Although we could not assure that the floor eggs were laid by the same hens, some studies showed that floor and nest eggs were consistently laid by the same floor or nest laying hens (Sherwin and Nicol, 1993; Cooper and Appleby, 1996; Kruschwitz et al., 2008; Zupan et al., 2008). Still, the factors that motivate the floor-nesting behavior are unclear: genetic differences (McGibbon, 1976), social dominance (Sherwin and Nicol, 1993), or simply because they do not want to use colony nest (Zupan et al., 2008).

Including 1.5% experienced hens did not impact incidence of floor eggs. However, caution should be taken when drawing conclusions on the effectiveness of including experienced hens because we did not evaluate any inclusion rate other than 1.5%. This result suggests that using experienced hens may not be an effective means to stimulate the motivation of young hens for using colony nests in aviary systems. Cooper and Appleby (1995) evaluated whether laying hens from conventional cages, deprived of resources that they had never experienced, would present similar level of motivation to use littered nests as those with prior experience, and found that the nest-seeking behavior was independent of prior experience of nesting cues. In an experiment to evaluate nesting behavior and gregarious nesting, Riber (2010) suggested that young inexperienced hens visited the same colony nest as frequently as the experienced hens. In addition, as the young hens gained experience they tended to rely more on their own experience in selection of nest. Richard-Yris and Leboucher (1987) evaluated whether the kinetics of maternal behavior could be inducted in successive experiments and found that the maternal behavior emerged gradually (significant day effect), but no difference was found between naïve hens and hens having already had a first induction experience.

On the other hand, managing the litter floor access during the oviposition time showed marked reduction of 93% in weekly percentage of floor eggs and 89% reduction in eggs per hen housed as of 76 WOA in the present study. Campbell et al. (2016a) evaluated the litter area usage in a commercial aviary (Lohmann White) and reported that the hens performed dust bathing throughout the day with peak dust bathing activity in the afternoon and late morning. This outcome is expected because in the morning period the hens are highly motivated to explore colony nests and lay their eggs, and oviposition with pre-laying activity can last 3.5 to 4.5 h after the lights come on (Hunniford et al., 2014, 2017). Therefore, dust bathing during oviposition period (early to mid-morning) is less critical due to its lower motivation priority.

### Hens Outside the System at Night, BW and BW Uniformity

The percentage of birds remaining on the litter floor declined beginning at 21 WOA. This outcome presumably arose from the birds becoming more “trained” to the lighting program (“calling” them back to the system at night) and the aviary system.

The biggest concern was with the hens in the PLA regimen because if they remained outside the system at night they could only have access to feed and water after the doors were reopened the next day. However, the percentage of hens in the PLA regimen that remained on the litter area at night were quite minimal (0.010 ± 0.001% or averaging 1 per 10,000 hens), and the regimen did not affect BW or BW uniformity of the flock. Very low percentage of hens in the FLA regimen (0.040 ± 0.002%) were observed in the litter area before the lights came on. It was not clear if the presence of these hens stemmed from their natural preference or was induced by the walking sound or night light used by the caretaker when counting the hens. Campbell et al. (2016b) reported that the majority of hens in an aviary facility voluntarily returned to the system in the evening and the rest remained on the litter floor until the doors were reopened the next day.

### Mortality Rate

There was no evidence that managing litter access or including 1.5% experienced hens affected the flock mortality. The overall average cumulative mortality rate in the current study (14.3 ± 0.4%) was higher than the reference value for Dekalb White hens (Hendrix Genetic Company, 2018) in alternative housing systems (95.2% livability or cumulative mortality of 4.8% by 76 WOA). Studies with aviary systems have reported cumulative mortality of 6.51 to 6.68% by 70 WOA (Long et al., 2016), 3.2% by 52 WOA (Sirovnik et al., 2018), 6.7 to 16.3% by 80 WOA (Abrahamsson and Tauson, 1995), 5 to 20% by 70 WOA (Nicol et al., 2006), and 11.5% by 78 WOA (Karcher et al., 2015). Therefore, the cumulative mortality rate observed in the current study by and large paralleled those of the reported field studies.

### Litter Conditions

The conditions of the litter were affected by managing the litter floor access, and the main reason for the impact is the extended time (approximately 6 h during light period) of litter access in the FLA regimen. Moisture content in the FLA regimen averaged 54% higher than that in the PLA regimen. Litter depth in FLA averaged 130% higher than that in PLA, which translates to the additional amount of litter removed during cleaning period. Accordingly, litter texture was different between PLA and FLA regimens in that litter in FLA was mostly caked during the first 3 mo, hindering the hens’ dust bathing activities due to lack of loose litter. The increased caking presumably arose from the thicker litter being more difficult to be dried by the ventilation air.

Litter accumulation on the floor varied with time and the litter access management (PLA vs. FLA). In this study, litter accumulation rate was higher in FLA (ranging from 0.44 to 1.15 mm/d) than in PLA (ranging from 0.22 to 0.31 mm/d). In a similar housing system, Lohmann SL White hens having 9.75 h of access to litter area per day showed a litter depth increase rate of 0.12 cm/wk (0.17 mm/d) (Zhao et al., 2013). The average maximum litter depth in FLA and PLA was 6.3 and 2.2 cm, respectively. Campbell et al. (2016a) reported that litter depth did not exceed 6.6 cm during the whole laying cycle with PLA in a similar commercial aviary house.

To avoid the excessive litter accumulation in the FLA regimen, it would be necessary to increase the frequency of litter removal from once every 4 mo to once a month, which means the need to lock the hens in the system more often and extra labor for the cleaning. The amount of as-is litter removed averaged 130% higher (or 2.3 times) in FLA than in PLA, directly resulting from the extra time of litter floor access for the FLA regimen.

Total bacteria concentrations of litter samples were comparable to the results reported by Zhao et al. (2016), who found the bacteria concentration of litter samples (as is) in aviary system to be 9.2 ± 0.8 log CFU/g. No difference in bacteria concentration between PLA and FLA regimens was found in this study. This test was performed only at the end of the experiment, when no significant difference in litter moisture between the regimens was detected (20.6 ± 1.2% for FLA and 19.6 ± 1.2% for PLA, *P* = 0.57) and texture of the litter in both regimens was mostly loose.

### Environmental Conditions

There were several cold days during the experimental period, but with the supplemental heat, the indoor temperature was maintained above 20°C most of the time. The indoor temperature increased during the summer and was close to the ambient temperature, indicating that the ventilation system was well managed to remove the excess heat produced by the laying hens. The PLA or FLA regimen did not affect the microclimate. Although the hens in FLA had approximately 6 h more to spend in the litter area than the hens in PLA, no differences in temperature or RH were observed. Mixing fans located in the litter area also contributed to improving the heat distribution. The indoor RH was relatively stable, averaging 65 to 75% during cold season and 55 to 65% during warm season. The seasonal differences in RH were mostly attributed to the season-dependent ventilation rate (lower in winter and higher in summer). A similar pattern was observed by Zhao et al. (2013).

Indoor CO_2_ concentration ranged from 695 to 4,132 ppm (by volume), inversely related to ambient temperature. Analogous to RH, CO_2_ concentration was lowest under the warmer weather and the associated maximum ventilation rate, and highest during the cold weather and the associated minimum ventilation rate.

Management of littered floor has a significant effect on ammonia concentration. Appropriate ventilation rate can reduce litter moisture content and thus ammonia release into the air (Xin et al., 2011). During the warm weather period, increased ventilation dried the litter more effectively, which reduced the ammonia generation, and further diluted its concentration. On the other hand, ammonia concentration peaked during the cold weather due to the minimum ventilation. Managing the access to litter area affected litter accumulation on the floor, moisture content, and consequently the ammonia release. Hence, it was not surprising that the FLA regimen showed the highest values in all these variables (litter/manure amount, depth, moisture content, and NH_3_ concentration).

Caked litter is detrimental to hens’ health and welfare because a) it has higher moisture content, and thus a source of higher ammonia release; and b) caked litter makes it difficult for the hens to perform dust bathing, which is one of the main purposes for providing the litter area in CF systems. On the other hand, hens in PLA deposited manure onto the manure belts in the system from 05:00 am to 10:50 am (lights on to system opening), while the litter area was being completely exposed (without hens), which facilitated the manure drying by the ventilation air. This process presumably reduces ammonia volatilization by reducing decomposition rate of uric acid in the manure (Sorefferle, 1965; Molloy and Tunney, 1983; Brinson et al., 1994). In addition, manure on the belts was removed much more frequently (every 3 d) than manure deposited on the litter floor (3 times per year).

During the cold weather, average ammonia concentration exceeded 25 ppm, the 8-h exposure threshold for workers recommended by NIOSH. Ammonia concentration exceeded 25 ppm in January 2017 in both PLA and FLA, whereas in February 2017 and January 2018 the exceedance occurred only in FLA. Although the month of December (2017) registered very low ambient temperature (minimum of –29°C), the ammonia concentration data were collected on a mild day (15°C) and proper ventilation rate was applied. Hence, the snapshot measurement of the lower ammonia concentrations was likely not reflective of the actual levels in the cold weather.

### Welfare Status

This study did not find any effect of the litter access management (*P* > 0.05) on the welfare status of the laying hens by 72 WOA. However, occurrence of poor plumage condition, keel bone deformation, long claws, and foot pad dermatitis were quite frequent. We did not find statistical evidence that including 1.5% experienced hens would affect the laying hens’ welfare, with the exception of the claw length (*P* = 0.01). Nonetheless, we speculate that this exception was not a cause–effect relationship, but more related to the sampling.

The overall feather coverage score of laying hens was relatively low (approximately 5 out of 14), meaning a good feather condition at 72 WOA. This outcome agreed with previous studies that evaluated welfare conditions of laying hens in conventional and alternative housing systems (Rodenburg et al., 2008; Blatchford et al., 2016). Plumage condition is associated with the hens’ capability of thermoregulation, and a poor feather condition will affect the hens’ welfare, increase the loss of body heat, and feed energy intake to maintain homeostasis in cold weather (Sarica et al., 2008).

The incidence of keel bone deformity was moderately high, with approximately 57% of the hens showing keel bone deformation. Different studies reported frequency of keel bone deformation varying from 56 to 97% (Rodenburg et al., 2008; Käppeli et al., 2011; Wilkins et al., 2011). In CF systems, fall and collisions with perches and other parts of the housing system (Stratmann et al., 2015), and the extended perching behavior with long-term pressure on the keel bone (Tauson and Abrahamsson, 1994) are assumed to be the main causes for the high incidence of keel bone damage. Keel bone deformation or fracture has been shown to be associated with pain (Nasr et al., 2012b), decrease egg production (Nasr et al., 2012a), and elevate mortality (McCoy et al., 1996).

In this study, 35% of the hens showed some extent of foot pad disorder, which was consistent with the results revealed by Heerkens et al. (2016) that the prevalence of dermatitis ranged from 36.5 to 38.5% in ISA Brown and Dekalb White hens at 29 to 49 WOA. Foot pad disorders are mostly caused by prolonged pressure load on the foot pads when perching, standing on wire floor, grabbing (Weitzenbürger et al., 2006), and can be particularly painful to hens (Tauson and Abrahamsson, 1994).

Prevalence of excessive claw growth was observed in this study (78%). It can lead to easy break off, causing bleeding and possibly infection (Lay et al., 2011). Vits et al. (2005) reported that the claw length was affected by housing systems because of the different options of shortening devices used. Although not quantified, we did observe few incidences of hens with their claws stuck in the structure of the system. It can cause serious injury if the hens are not attended in a timely manner.

## CONCLUSIONS

FLA of the aviary housing system showed a number of shortcomings when compared with PLA, including much higher incidence of floor eggs, higher ammonia concentration, more presence of caked litter, and greater accumulation of manure on the floor which necessitates more frequent removal from the barn. No difference was detected between FLA and PLA in hen welfare, mortality, BW, BW uniformity, or litter bacteria concentration. Inclusion of experienced hens (1.5%) in a young flock did not show benefit of inducing nest-laying behavior, and the young hens learned to return to the system at night quickly.
